# Plaque-Like Dermatofibroma: Case Report of a Rare Entity

**DOI:** 10.3390/dermatopathology8030038

**Published:** 2021-08-01

**Authors:** Sara Moradi, Laila Mnayer, Jonathan Earle, Alex C. Cech, Torsten Ehrig

**Affiliations:** 1 Department of Pathology and Laboratory Medicine, Hartford Hospital, Hartford, CT 06106, USA; sara.moradi@hhchealth.org (S.M.); Laila.Mnayer@hhchealth.org (L.M.); Jonathan.Earle@hhchealth.org (J.E.); 2 Hartford Health Care Medical Group, Farmington, CT 06032, USA; alex.cech@hhchealth.org

**Keywords:** giant dermatofibroma, plaque-like dermatofibroma, multiple clustered dermatofibromas, plaque-like myofibroblastic tumor, histopathology

## Abstract

A case of a well-demarcated plaque measuring 11 cm without satellites of several years’ duration is presented. It showed typical histologic findings of dermatofibroma, prompting a diagnosis of plaque-like dermatofibroma. The relationship to multiple clustered dermatofibromas and plaque-like myofibroblastic tumor is discussed.

## 1. Introduction

Giant dermatofibromas have been defined by a size of at least 5 cm [1] and typically have a nodular and often pedunculated appearance. A subtype of giant dermatofibroma with a less elevated appearance has been termed “plaque-like dermatofibroma”. Only a few cases of this entity have been reported. We here describe an additional case and discuss its relationship to cases that have been termed “multiple clustered dermatofibromas” and “plaque-like myofibroblastic tumor”.

## 2. Case Report

A 43-year-old female with a past medical history of psoriasis and psoriatic arthritis who recently had been taking immunomodulatory medications presented with a long-standing large plaque on her mid back.

**Figure 1:**
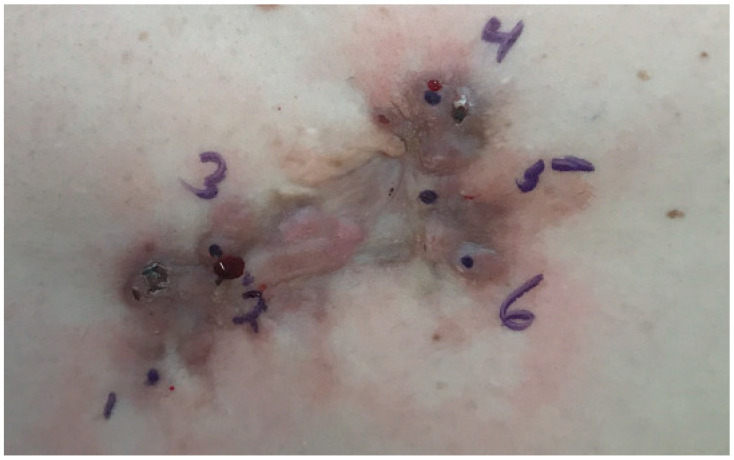
A plaque with irregular brown pigmentation.

**Figure 2:**
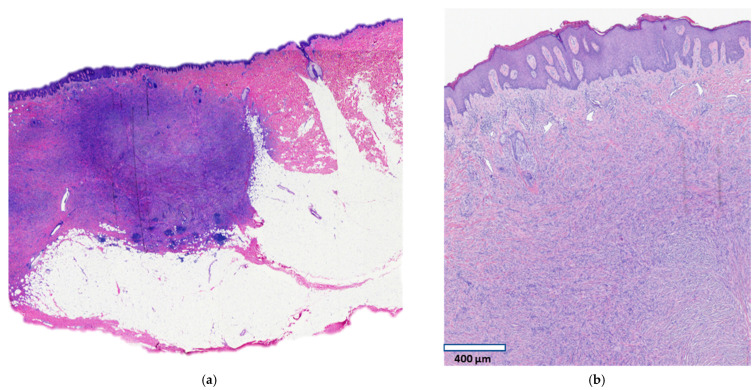

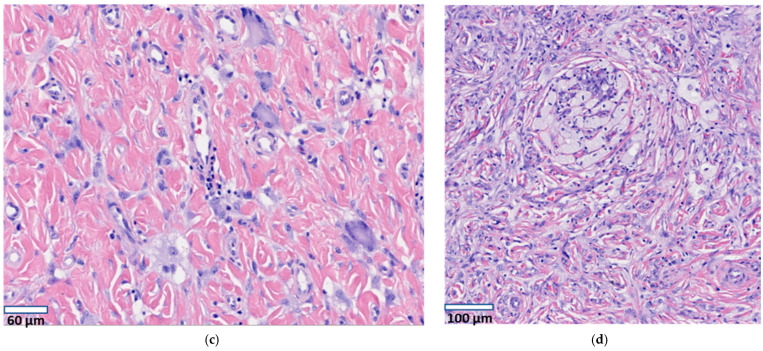
Histopathology of plaque-like dermatofibroma. Well-demarcated infiltrate of spindle cells in the dermis and upper subcutis (**a**,**b**) with overlying epidermal hyperplasia ((**b**), scale bar: 400 µm), multinucleated cells ((**c**), scale bar: 60 µm), and foam cells ((**d**), scale bar: 100 µm).

A focus of the patient’s psoriasis had been biopsied fifteen years earlier at the same location as the current plaque, after which the wound healed with a nodular, painful scar. Since that time, the lesion has been slowly increasing in size and has become more symptomatic with pain and pruritus. Clinically it was suspicious for a keloidal scar. The patient denied any specific treatments directed at the lesion. Examination revealed an irregularly pigmented plaque of irregular outlines measuring 11 × 5 cm (Figure 1). Six punch biopsies demonstrated typical findings of dermatofibroma. The plaque was excised surgically. Histology of the punch biopsies and of the surgical specimen revealed a moderately cellular infiltrate of bland spindle cells in the dermis and upper subcutis (Figure 2a) in a haphazard to focally storiform arrangement. The subcutis was involved by pushing broad aggregates of spindle cells, without infiltration between adipocytes (honeycomb pattern) (Figure 2a). The overlying epidermis demonstrated hyperplasia (Figure 2b), and multinucleated cells (Figure 2c) and foam cells (Figure 2d) were present. These histologic features are typical of a dermatofibroma and exclude a diagnosis of dermatofibrosarcoma protuberans. A few foci of follicular induction in the form of nests of basaloid cells with peripheral palisading situated at the undersurface of the epidermis were identified. Immunohistochemical stains showed expression of Factor XIIIa and no expression of CD34 or WT1, further excluding a diagnosis of dermatofibrosarcoma protuberans. Next-generation sequencing including RNA expression analysis was performed following manual microdissection of lesional tissue using a customized platform (ArcherDx, Boulder, CO, USA). No clinically reportable sequence alterations, copy number changes, or fusions were identified by targeted next-generation sequencing, however, the RNA expression visualization showed increased expression of FGFR1, and sequencing showed a variant of uncertain significance (VUS) in FGFR3.

## 3. Discussion

Among the many variants of dermatofibroma, the plaque-like lesion reported here is best classified within a group of variants that have variously been reported under the diagnoses “plaque-like dermatofibroma” (PLDF) [2,3,4,5,6,7], “multiple clustered dermatofibromas” (MCDF) [8,9,10,11,12,13,14,15,16,17,18,19,20,21,22], and “plaque-like myofibroblastic tumor” (PLMT) [23,24,25]. Lesions that have been published under these three diagnoses show the histopathologic features of dermatofibroma and are characterized by similar or related clinical findings (predilection for the lumbar-hip-thigh area and slow growth over many years/decades), and they may therefore represent variants of a disease spectrum rather than different entities. At one end of the clinical spectrum are cases where papules predominate [19,20,21,22], and at the other end are cases that consist of a single, well-demarcated plaque [2,23,25]. The case reported here is an example of the latter category. Most cases occupy an overlapping middle ground, consisting of multiple papules/nodules that coalesce into a centrally located plaque, and such cases have been reported both under the heading of PLDF [2,3,4,5,6,7] and MCDF [8,9,10,11,12,13,14,15,16,17,18], and in some cases a description of “plaque-like dermatofibroma with satellites” was chosen [6,7]. The condition termed “plaque-like myofibroblastic tumor” (PLMT) occurs in infancy and similarly consists of multiple clustered papules often coalescing into a plaque. These cases show histopathologic findings that are typical of dermatofibroma, but they were originally thought to represent a different entity because they demonstrate diffuse immunohistochemical positivity for smooth muscle actin. This was interpreted as myofibroblastic differentiation and prompted the designation “plaque-like myofibroblastic tumor” (PLMT) [23]. However, dermatofibromas often show focal and, in some cases, diffuse reactivity for smooth muscle antigen [26], and it has therefore been suggested that PLMT may represent the childhood equivalent of MCDF/PLDF rather than a separate entity [4,25]. In general, we agree with those authors who have suggested that MCDF, PLDF, and PLMT represent variations of a single disease entity [4,5,9,19,25].

Of note, some cases of clustered or plaque-like dermatofibromas appear to be different from the MCDF/PLDF/PLMT group. Most notable among these are four case reports of patients who also had multiple clustered or plaque-like dermatofibromas that, however, differed by their eruptive time course and, in three cases, by coincidence with the administration of immunosuppressive medication [27,28,29,30]. Our patient clearly does not fit into this group because her plaque exhibited very slow growth, and she had taken immunomodulatory medication only in the recent past.

By history, the plaque of our patient was preceded by trauma in the form of a biopsy. This may have been coincidental, as trauma has been observed occasionally [4,6,13] but not consistently as a cause of MCDF, PLDF, or PLMT.

The origin of dermatofibromas is unclear. A cause related to an immune process or to immunosuppressive medication has been suggested based on the observation that a subgroup of dermatofibromas occurring as multiple, widespread eruptive lesions are often associated with an autoimmune disease, most commonly lupus erythematosus [31,32]. In these patients, the dermatofibromas show a wide anatomic distribution without clustering. It is not clear how this group of patients relates to the usual single dermatofibromas or to the above settings of MCDF, PLDF, and PLMT, which in general are not associated with an immune-mediated disease. Some cases of benign fibrous histiocytomas/dermatofibromas have been found to carry gene fusions involving protein kinase C isoenzymes PRKCA, PRKCB, and PRKCD and occasionally involving the ALK gene [33,34], but for the majority of cases the genetic basis is unknown. Furthermore, it is unknown what factors limit the growth of dermatofibromas and which factors are responsible for the development of giant dermatofibromas. We applied two sequencing panels that detect gene mutations or fusions commonly encountered in neoplastic disease (including protein kinase C isoenzymes PRKCA and PRKCB as well as ALK, but not PRKCD) to our plaque-like dermatofibroma but did not find a genetic alteration. In an mRNA expression platform, we identified an overexpression of FGFR1 (fibroblast growth factor receptor 1). This may be related to the increase in expression of FGFR2 and FGFR4 that was reported to have been detected in dermatofibromas by immunohistochemistry [35]. Overexpression of FGFRs results in the activation of MAPK and PI3K/Akt pathways and is found in benign and malignant tumors [36,37,38]; it is therefore possible that a similar growth-stimulatory mechanism is operative in dermatofibromas. Final classification of MCDF, PLDF, and PLMT awaits further support from molecular data to help determine whether they represent separate entities or a single disease process with variable clinical features.

## References

[1] Requena L., Fariña M.C., Fuente C., Piqué E., Olivares M., Martín L., Yus E.S. (1994). Giant dermatofibroma. A little-known clinical variant of dermatofibroma. J. Am. Acad. Dermatol..

[2] Panicker V.V., Dharmaratnam A.D., Seethalekshmy N.V. (2017). Plaque-like giant dermatofibroma: A case report. J. Cutan. Aesthet. Surg..

[3] Micantonio T., Fargnoli M.C., Peris K. (2005). Giant Dermatofibroma Appearing During Pregnancy. Acta Derm. Venereol..

[4] Leow L.J., Sinclair P.A., Horton J.J. (2008). Plaque-like dermatofibroma: A distinct and rare benign neoplasm?. Australas. J. Dermatol..

[5] Lozano Masdemont B., Campos Domínguez M., Gómez-Recuero Muñoz L., Bergón Sendín M., Parra Blanco V., Suárez Fer-nández R. (2016). Multiple clustered dermatofibroma: A rare variant of plaque-like dermatofibroma. G Ital. Dermatol. Venereol..

[6] Findeis S., Lynch M.C., Sceppa J., Helm K.F. (2017). Plaque-like dermatofibroma with satellitosis. Dermatol. Surg..

[7] Avila C., Lause M., McIntyre M., Rzepka P.V., Chung C., Trinidad J. (2019). Plaque-like dermatofibroma with satellitosis in a young woman. Int. J. Dermatol..

[8] Shaheen B., Saldanha G., Calonje E., Johnston G.A. (2013). Multiple clustered dermatofibromas (fibrous histiocytomas): An atypical clinical variant of dermatofibroma. Clin. Exp. Dermatol..

[9] Higaki-Mori H., Yoshida Y., Hisaoka M., Nishigori C., Shindo M., Yamamoto O. (2019). Unusual Congenital Multiple Clustered Dermatofibroma: First Reported Case on the Face. Acta Derm. Venereol..

[10] Finch J., Berke A., McCusker M., Chang M.W. (2014). Congenital multiple clustered dermatofibroma in a 12-year-old girl. Pediatr. Dermatol..

[11] Gershtenson P.C., Krunic A.L., Chen H.M. (2009). Multiple clustered dermatofibroma: Case report and review of the literature. J. Cutan. Pathol..

[12] Soon S.L., Howard A.K., Washington C.V. (2003). Multiple, clustered dermatofibroma: A rare clinical variant of dermatofibroma. J. Cutan. Med. Surg..

[13] Berbis P., Benderitter T., Perier C., Frey J., Privat Y. (1988). Multiple Clustered Dermatofibromas. Dermatology.

[14] De Unamono P., Carames Y., Fernandez-Lopez E., Hernández-Martín A., Peña C. (2000). Congenital multiple clustered dermatofibroma. Br. J. Dermatol..

[15] Pinto-Almeida T., Caetano M., Alves R., Selores M. (2013). Congenital multiple clustered dermatofibroma and multiple eruptive dermatofibromas—Unusual presentations of a common entity*. Anais Brasileiros de Dermatologia.

[16] Espiñeira-Carmona M.J., Salazar-Nievas M., Girón-Prieto M.S., Aneiros-Fernández J., Buendía-Eisman A., Arias-Santiago S.A. (2013). Multiple clustered dermatofibromas. Eur. J. Dermatol..

[17] Sanli H., Akay B.N., Heper A.O. (2009). Congenital multiple clustered dermatofibroma: Dermatoscopic findings. Eur. J. Dermatol..

[18] Mitri F., Haenssle H., Enk A., Toberer F. (2020). Congenital multiple clustered dermatofibroma on the abdomen. Br. J. Dermatol..

[19] Reynolds H., Perry A., Satter E.K. (2014). Multiple clustered and focally atrophic dermatofibromas (DF). Dermatol. Online J..

[20] Veraldi S., Bocor M., Gianotti R., Gasparini G. (1991). Multiple Eruptive Dermatofibromas Localized Exclusively to the Buttock. Int. J. Dermatol..

[21] Komforti M., Jewell J., Pavlis M. (2016). Multiple Clustered Dermatofibromas Associated with Pulmonary Arterial Hypertension. Am. J. Dermatopathol..

[22] Rognstad Ø.B., Haas N., Sterry W., Astner S. (2009). Multiple clustered dermatofibroma with overlying sebaceous hyperplasia. J. Dtsch. Dermatol. Ges..

[23] Clarke J.T., Clarke L.E., Miller C., Helm K.F., Zaenglein A.L. (2007). Plaque-like myofibroblastic tumor of infancy. Pediatr. Dermatol..

[24] Moulonguet I., Biaggi A., Eschard C., Durlach A., Stock N., Delanoé P., Coussirat-Voreaux M.-F., Fraitag S. (2017). Plaque-Like Myofibroblastic Tumor: Report of 4 Cases. Am. J. Dermatopathol..

[25] Marqueling A.L., Dasher D., Friedlander S.F., McCalmont T.H., Frieden I.J. (2013). Plaque-Like Myofibroblastic Tumor: Report of Three Cases. Pediatr. Dermatol..

[26] Berklite L., Ranganathan S., John I., Picarsic J., Santoro L., Alaggio R. (2020). Fibrous histiocytoma/dermatofibroma in children: The same as adults?. Hum. Pathol..

[27] Caldarola G., Bisceglia M., Pellicano R. (2012). Multiple eruptive plaque-like dermatofibromas during anti-TNFα treatment. Int. J. Dermatol..

[28] Viseux V., Chaby G., Agbalika F., Luong M., Chatelain D., Westeel P.-F., Denoeux J., Lok C. (2004). Multiple Clustered Dermatofibromas on a Superficial Venous Thrombosis in a Kidney-Transplanted Patient. Dermatology.

[29] Ruiz-Villaverde R., Diaz-Martinez M.A., Sancez-Cano D. (2017). Multiple clustered dermatofibromas following Ustekimumab treatment for psoriasis vulgaris. Sultan Qaboos Univ. Med. J..

[30] Bhabha F.K., Magee J., Ng S.Y., Grills C.E., Su J., Orchard D. (2014). Multiple clustered dermatofibroma presenting in a segmental distribution. Australas. J. Dermatol..

[31] Katsuoka K., Happle R., Hoffmann R., Niiyama S. (2002). Multiple eruptive dermatofibromas: A review of the literature. Acta Derm. Venereol..

[32] Beatrous S.V., Riahi R.R., Grisoli S.B., Cohen P.R. (2017). Associated conditions in patients with multiple dermatofibromas: Case reports and literature review. Dermatol. Online J..

[33] Płaszczyca A., Nilsson J., Magnusson L., Brosjö O., Larsson O., von Steyern F.V., Domanski H., Lilljebjörn H., Fioretos T., Tayebwa J. (2014). Fusions involving protein kinase C and membrane-associated proteins in benign fibrous histiocytoma. Int. J. Biochem. Cell Biol..

[34] Walther C., Hofvander J., Nilsson J., Magnusson L., Domanski H.A., Gisselsson D., Tayebwa J., Doyle L.A., Fletcher C.D.M., Mertens F. (2015). Gene fusion detection in formalin-fixed paraffin-embedded benign fibrous histiocytomas using fluorescence in situ hybridization and RNA sequencing. Lab. Investig..

[35] Ishigami T., Hida Y., Matsudate Y., Murao K., Kubo Y. (2013). The involvement of fibroblast growth factor receptor signaling pathways in dermatofibroma and dermatofibrosarcoma protuberans. J. Med. Investig..

[36] Hafner C., Hartmann A., Van Oers J.M.M., Stoehr R., Zwarthoff E.C., Hofstaedter F., Landthaler M., Vogt T. (2007). FGFR3 mutations in seborrheic keratoses are already present in flat lesions and associated with age and localization. Mod. Pathol..

[37] Chakravarty D., Gao J., Phillips S.M., Kundra R., Zhang H., Wang J., Rudolph J.E., Yaeger R., Soumerai T., Nissan M.H. (2017). OncoKB: A Precision Oncology Knowledge Base. JCO Precis. Oncol..

[38] Zhou W.-Y., Zheng H., Du X.-L., Yang J.-L. (2016). Characterization of FGFR signaling pathway as therapeutic targets for sarcoma patients. Cancer Biol. Med..

